# Regulation of phosphoglycerate kinase 1 and its critical role in cancer

**DOI:** 10.1186/s12964-023-01256-4

**Published:** 2023-09-18

**Authors:** Kexin Zhang, Lixue Sun, Yuanyuan Kang

**Affiliations:** Department of Emergency and Oral Medicine, School and Hospital of Stomatology, China Medical University, Liaoning Provincial Key Laboratory of Oral Diseases, 117 North Nanjing Street, Heping Area, Shenyang, 110002 People’s Republic of China

**Keywords:** PGK1, Aerobic glycolysis, Autophagy, EMT, Tumorigenesis

## Abstract

Cells that undergo normal differentiation mainly rely on mitochondrial oxidative phosphorylation to provide energy, but most tumour cells rely on aerobic glycolysis. This phenomenon is called the "Warburg effect". Phosphoglycerate kinase 1 (PGK1) is a key enzyme in aerobic glycolysis. PGK1 is involved in glucose metabolism as well as a variety of biological activities, including angiogenesis, EMT, mediated autophagy initiation, mitochondrial metabolism, DNA replication and repair, and other processes related to tumorigenesis and development. Recently, an increasing number of studies have proven that PGK1 plays an important role in cancer. In this manuscript, we discussed the effects of the structure, function, molecular mechanisms underlying PGK1 regulation on the initiation and progression of cancer. Additionally, PGK1 is associated with chemotherapy resistance and prognosis in tumour patients. This review presents an overview of the different roles played by PGK1 during tumorigenesis, which will help in the design of experimental studies involving PGK1 and enhance the potential for the use of PGK1 as a therapeutic target in cancer.

Video Abstract

**Supplementary Information:**

The online version contains supplementary material available at 10.1186/s12964-023-01256-4.

## Introduction

Cancer is currently one of the leading causes of death worldwide. Despite the significant progress that has been made in cancer diagnosis and treatment, cancer remains a major health problem worldwide. According to the latest data from the WHO, the global cancer incidence increased by 50% before 2020, and it is predicted that there will be 15 million new cancer patients every year. In 2022, there were approximately 4,820,000 new cancer cases and 3,210,000 cancer-related deaths in China, and cancer has become a major public health problem [1].

Cancer cells can adapt to survive under unique conditions, such as the lack of O_2_, by reprogramming their metabolic mechanism to meet particular needs. Compared with normal cells that rely on oxidative phosphorylation (OXPHOS) to generate ATP, cancer cells can use aerobic glycolysis to survive in conditions of fluctuating oxygen tension due to the inconstant haemodynamics of distant blood vessels. Under the condition that the same molecular weight of glucose is consumed, cancer cells consume less oxygen content and produce less ATP via aerobic glycolysis than normal cells. The TCA cycle of aerobic glycolysis is shortened, and intermediates are used for substrate cycle substrate synthesis. Lactate, which is the principal end product of aerobic glycolysis, also promotes the development of cancer via multiple pathways [2]. Glycolytic enzymes enable the switch from oxidative phosphorylation to aerobic glycolysis and also function as protein kinases and transcription factors [3]. In the past few years, the role of glycolytic enzymes in cancer metabolism has attracted the attention of many researchers, and these enzymes have been identified as potential targets for cancer treatment.

Phosphoglycerate kinase (PGK) is a central glycolytic enzyme that is associated with the survival of every organism, and mutations in PGK result in a number of metabolic disorders, including mental retardation, neurological disorders and rhabdomyolysis [4]. The two isoforms of PGK, namely, PGK1 (Gene ID: 5230) and PGK2 (Gene ID: 5232), perform similar functions and have similar structures in humans; these proteins are composed of 417 amino acids with 87–88% sequence identity and have a molecular mass of approximately 45 kDa [5]. However, PGK1 and PGK2 have different expression patterns. PGK2 is encoded by an autosomal gene and is uniquely expressed in meiotic and postmeiotic spermatogenic cells, while PGK1 is located on the X-chromosome, is broadly expressed in most cell types, and plays a rate-limiting role in the second stage of glycolysis in the regulation of energy production and redox balance [6]. In addition to regulating glycolytic metabolism, PGK1 has many characteristics of oncogenes, promotes tumour cell proliferation, migration and invasion and is involved in the initiation of autophagy and DNA replication in cancer cells [7]. The role of PGK1 in cancer has been thoroughly studied. Thus, this paper reviews the role of PGK1, the molecular mechanisms underlying PGK1 expression and activity in cancer, and the prospects for the use of PGK1 as a new diagnostic biomarker and therapeutic target in cancer and drug resistance.

## Structure and basic function of PGK1

**Fig. 1:**
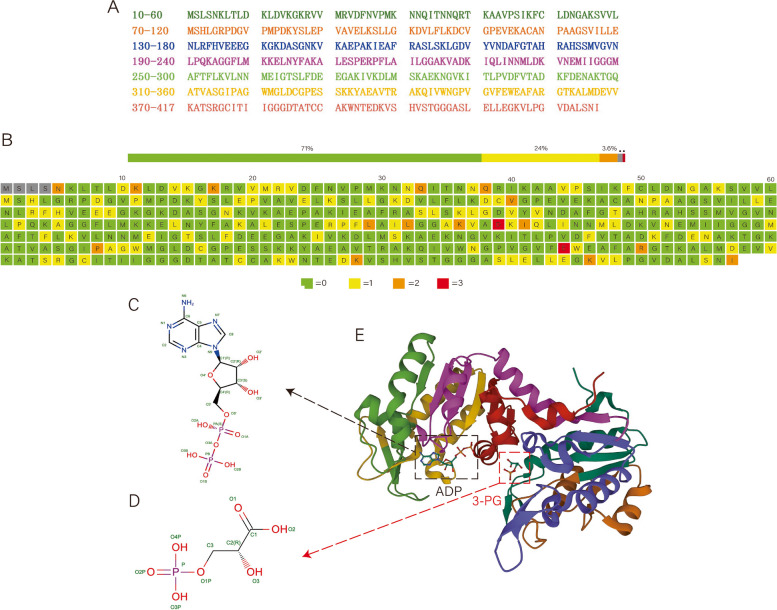
Amino acid sequence and structure of PGK1. **A** The Amino acid sequence of PGK1. **B** The Residue-property plots of PGK1 (green = 0, yellow = 1, orange = 2 and red = 3 or more). **C** 2D Diagram of ADP (C10 H15 N5 O10 P2). D. 2D Diagram of 3-PG (C3 H7 O7 P). **E** The structure of PGK1 (PDB: 2XE7, open conformation)

The protein structure of PGK1 is well understood [8]. PGK1 is a monomeric enzyme that is composed of 417 amino acids, and it contains two nearly equal-sized α-helical domains corresponding to the N- and C-termini of the protein, which form a characteristic bilobed structure [9]. 1,3-Bisphosphoglycerate (1,3-BPG) binds to the N-terminal domain, and ADP binds to the C-terminal domain of PGK1 [10]. This extended dual domain structure is connected by a "hinge bend'' conformational change [11]. Although the binding of either the 1,3-BPG or ADP substrate triggers a conformational change, domain closure occurs only when both substrates bind to PGK1, and this closure brings the two substrates into the proper proximity to allow phosphotransfer [12]. The amino acid sequence and tertiary structure of PGK1 are shown in Fig. 1.

PGK is highly conserved in structure, but the change in amino acid content at the N-terminus leads to structural differences in PGK adaptation to temperature and other factors in different environments [13]. PGK1 variants expressed in cancer cells show different catalytic activities compared with the conformational stability of natural enzymes [8]. As reported in Catalogue of Somatic Mutations in Cancer (COSMIC; http://cancer.sanger.ac.uk/cosmic), somatic mutations of PGK1 have been identified in different cancers [14]. Missense variants in the coding region are the main type of variant and lead to amino acid substitutions in the polypeptide sequence [15]. By regulating the ATP and 3-PG levels, PGK1 plays an important role in coordinating energy metabolism, biosynthesis and redox balance, so mutations in PGK1 can be responsible for changes in the metabolic spectrum in different cancers.

PGK1 performs a variety of physiologically related biochemical or biophysical functions and is a key metabolic enzyme. Under hypoxic conditions, PGK1 can may translocate from the cytoplasm to the mitochondrion, where it can act as a protein kinase and phosphorylate different protein substrates [16]. Interestingly, the protein kinase activity of PGK1 has been associated with the initiation of autophagy [17]. This enzyme may be secreted into the extracellular environment by tumour cells and act as a thiol reductase that regulates angiogenesis [18]. In addition, translocation of PGK1 to the nucleus is related to binding to alpha DNA polymerase [19].

## Implication of PGK1 in cancer progression

The expression and activity of PGK1 are strictly controlled by a balanced transcription network to ensure proper cell function and tissue development. Therefore, abnormal expression of PGK1 affects cell proliferation, differentiation, apoptosis, autophagy, migration, invasion, and DNA repair, which are closely associated with cancer initiation, development, metastasis, angiogenesis and drug resistance. A comprehensive understanding of the role of PGK1 in cancer progression will facilitate the development of better diagnosis and treatment strategies.

### Roles of PGK1 in cancer proliferation and initiation

The most remarkable characteristic of cancer is the constant proliferation and resistance to apoptosis of malignant cells. High intracellular expression of PGK1 leads to tumour cell proliferation. The oncogenic role of PGK1 is closely associated with promoting cell proliferation and inhibiting apoptosis in a variety of cancers, including liver cancer [7, 20] colon cancer [21], breast cancer [22] and GBM [16, 23]. PGK1 expression was shown to be significantly upregulated in multiple cancer tissues compared with corresponding nontumor tissues, which clearly indicates the oncogenic function of PGK1. The ability of PGK1 to promote tumour proliferation may be partly due to its involvement in the positive regulation of PTMs.

For instance, acetylation of PGK1 at the K323 site is an important regulatory mechanism for promoting its enzymatic activity as well as liver cancer cell proliferation and tumorigenesis [7]. In fact, blocking T255 O-GlcNAcylation of PGK1 decreases colon cancer cell proliferation, suppresses glycolysis, promotes the TCA cycle, and inhibits tumour growth in xenograft models [21]. Similar results have been reported in brain tumours [24], indicating the crucial role of PGK1 in driving cancer initiation by regulating PTMs.

However, PGK1 plays a negative role in cancer progression by suppressing tumour growth in other types of cancer. In prostate cancer, extracellular PGK1 has disulfide reductase activity against fibrinolytic enzymes, leading to elevated plasma levels of angiostatin, thereby inhibiting angiogenesis and tumour growth [18, 25]. In addition, the mechanism underlying PGK1-mediated tumour inhibition also includes the overexpression of PGK1 in Lewis lung cancer and the downregulation of COX-2 expression to reduce tumour growth in vivo [26]. This suggests that PGK1 can enhance the ability of immune cells to recognize and bind to antigens, thus enhancing cancer cell elimination.

Therefore, further research on more types of cancer and the high levels of PGK1 in/out of cells is needed to determine whether PGK1 is an inhibitor or activator of tumours, and these studies will support the accurate use of PGK1 therapy in the treatment of cancer.

### Roles of PGK1 in cancer angiogenesis

**Fig. 2:**
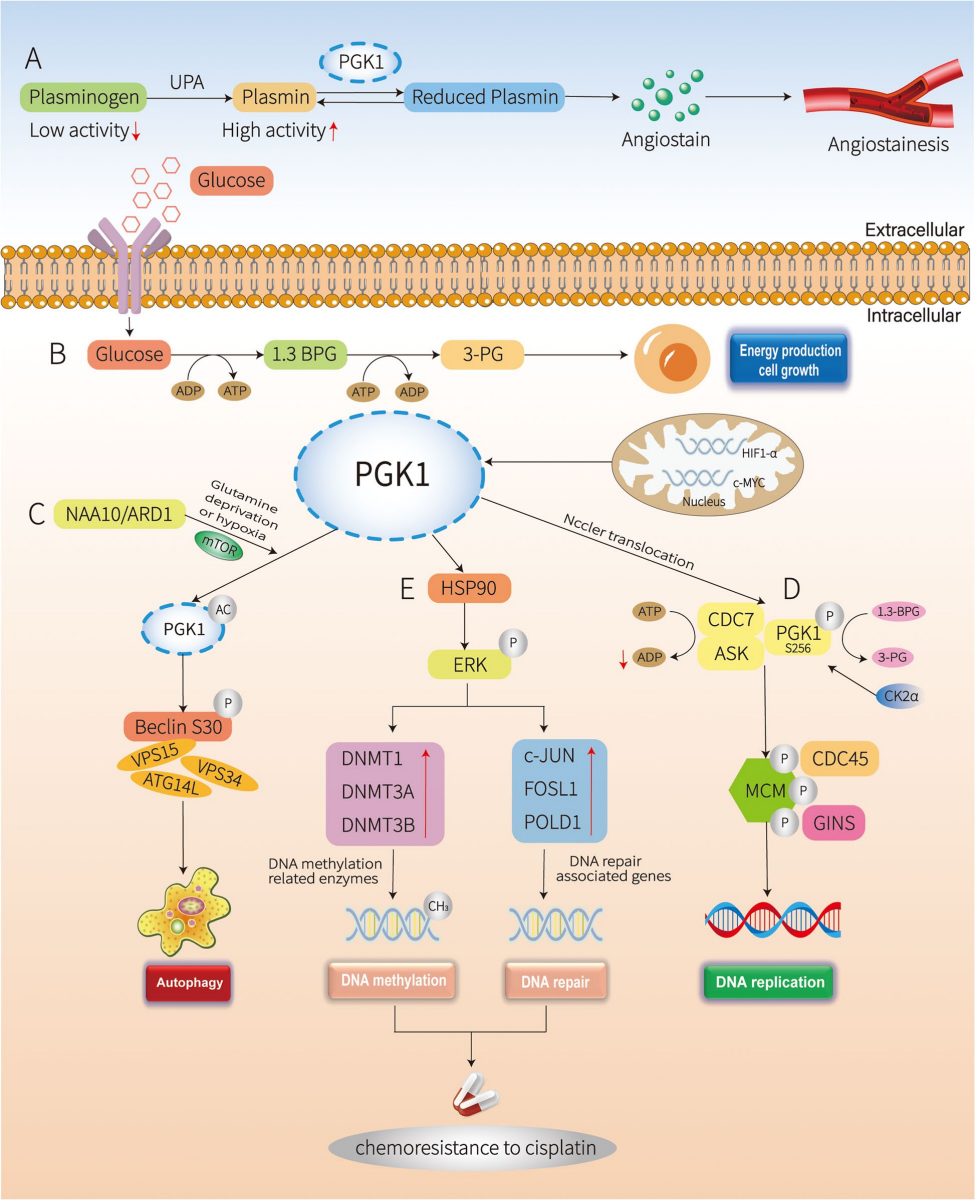
Molecular mechanism mediated by PGK1. PGK1 promotes tumorigenesis by regulating autophagy, DNA replication, while it can also inhibit tumor growth by inhibiting angiogenesis. **A** Extracellular PGK1 inhibits angiogenesis. **B** PGK1 has an essential role in metabolic reprogramming induced by c-Myc and HIF-1ɑ, leading to an enhanced Warburg effect and tumorigenesis. **C** PGK1-regulated autophagy initiation under glutamine deprivation and hypoxia. **D** PGK1 is a coactivator of transcription factors and is associated with DNA replication. **E** PGK1 is associated with DNA methylation and repair

Angiogenesis is one of the main mechanisms underlying PGK-mediated tumour growth and migration. High extracellular PGK1 levels suppress cancer malignancy by inhibiting angiogenesis. Intracellular PGK1 seems to play contradictory and even opposite roles compared with extracellular PGK1 in cancer occurrence. Extracellular PGK1 can function as a disulfide reductase, which generates angiostatin by increasing the reduction of disulfide bonds in plasmin (Fig. 2A) [25]. The formation of angiostatin from plasmin inhibits tumour growth by suppressing angiogenesis [27]. A recent study demonstrated that PGK1 expression is repressed by MVIH, a long noncoding RNA (lncRNA) that activates tumour-induced angiogenesis in hepatocellular carcinoma (HCC) [28]. Therefore, extracellular PGK1 plays an inhibitory role in tumour growth and metastasis under certain conditions, and it is essential for PGK1 to be properly balanced to promote cancer.

### Roles of PGK1 in cancer EMT

Epithelial-mesenchymal transformation (EMT) refers to the biological process in which epithelial cells transform into cells that exhibit a more mesenchymal phenotype. EMT has been previously reported to play an important role in embryonic development, wound healing and tumour metastasis [29]. The main characteristic of EMT is a reduction in the expression of cell adhesion molecules such as E-cadherin and the conversion of expression profiles from keratin expression to vimentin expression in the cytoskeleton [29].

Hypoxia can increase PGK1 expression, which promotes glycolysis, enhances stem cell-like properties and promotes EMT by activating AKT signalling in OSCC [30]. In NSCLC, GBP1 regulates erlotinib resistance via PGK1-mediated EMT signalling, proving that PGK1 plays a role in regulating EMT [31]. MMPs and E-cadherin are important markers in the EMT process. In prostate cancer, PGK1 has been demonstrated to promote MMP-2 and MMP-9 expression in normal fibroblasts (NSFs), resulting in induction of the phenotypic transformation of NSFs into cancer-associated fibroblasts (CAFs) [32]. In gastric cancer, nuclear PGK1 can inhibit E-cadherin expression [33]. These results indicate that PGK1 acts as an important regulator of EMT.

### Roles of PGK1 in cancer invasion and metastasis

Metastasis is a complex multistep pathological process that plays a crucial role in the incidence and mortality of cancer [34]. In the process of invasion and metastasis, cancer cells migrate and colonize secondary sites far from the primary tumour. Although considerable progress has been made in studying the mechanism underlying invasion and metastasis, how this process is regulated is still largely unknown.

In recent years, PGK1 proteins have been identified as critical regulators of tumour invasion and metastasis. The downregulation of PGK1 can obviously inhibit the invasion of breast cancer cells and reverse the EMT process. The PGK1 and HIF-1ɑ pathways stimulate the development and metastasis of breast cancer [35]. An important link between PGK1 expression and CXCR4/CXCL12 has been reported in prostate cancer cells, where overexpression of PGK1 increases the cell metastasis rate [18]. Collectively, these studies suggest that PGK1 is an effective target to prevent cancer invasion and metastasis as well as cancer progression.

## PGK1 functions in cancers

### PGK1 and glycolysis

Many original tumour sites have low concentrations of molecular oxygen, and this conditions is known as hypoxia. Most cancer cells, even in the presence of ample oxygen levels, predominantly generate adenosine triphosphate (ATP) by a high rate of glycolysis followed by the fermentation of lactate in the cytosol rather than by the oxidation of pyruvate in the mitochondria, which is the process used by most normal cells. This phenomenon, known as aerobic glycolysis or the Warburg effect, facilitates tumour cell growth [36, 37]. HIF-1ɑ serves as a transcription factor to promote glycolysis for the Warburg effect [38].

PGK1, a critical component of the glycolytic pathway, is a rate-limiting enzyme that catalyses the conversion of 1,3-BPG and ADP into 3-phosphoglycerate (3-PG) and ATP (Fig. 2B). The PGK1-catalysed reaction is the first ATP-yielding step of glycolysis and is essential for energy generation in most living cells, that is, via the glycolytic pathway in aerobes and the fermentation in anaerobes [39]. By controlling the ATP and 3-PG levels, PGK1 plays an important role in coordinating energy production with biosynthesis and redox balance. The expression of PGK1 is stimulated by several known oncogenes or transcription factors, such as Myc and HIF-1ɑ [40]. Myc is an important regulator that controls the activity of the PGK1 enzyme, and in ovarian cancer, Pim1 can influence cellular metabolic processes via the c-Myc-PGK 1 axis [41]. Myc-dependent PGK1 expression induces the overexpression of key glycolysis enzymes GLUT4, HK2 and LDHA to accelerate glycolysis and produce large amounts of ATP and lactate, thus promoting HCC proliferation and metastasis. Early studies have shown that PGK1 is directly regulated by HIF-1ɑ in cultured human colon carcinoma cells [42] and hepatoblastoma cells [43, 44]. In human colon cancer cells, PGK1 has been identified as a potential biomarker that reflects intracellular oxidative stress status, and antioxidants can inhibit the expression of HIF-1ɑ and PGK1 [42]. Yang et al. proposed that HIF-2ɑ, which is another hypoxia-inducible factor, could interact with PGK1, thus accelerating the invasion of breast cancer [45]. In addition, Yajuan Zhang [23] found that macrophage-associated PGK1 phosphorylation could promote aerobic glycolysis and tumorigenesis.

### PGK1 and autophagy

Autophagy is a process by which cells capture intracellular proteins, lipids, and organelles and deliver them to the lysosomal compartment for degradation [46]. Studies have shown that the protein kinase activity of PGK1 is directly or indirectly related to the regulation of autophagy [17]. NAA/ARD1 is the catalytic subunit of N-acetyltransferase (NAT) group A (NatA)1 and is involved in various cellular functions that regulate cell division, proliferation and tumorigenesis [47]. It has been experimentally proven that NAA10/ARD1-dependent PGK1 acetylation and PGK1-mediated Beclin1 S30 phosphorylation are required for glutamine deprivation- and hypoxia-induced autophagy and brain tumorigenesis (Fig. 2C). PRAS40 (encoded by AKT1S1) was primarily determined to be a binding protein of 14–3-3 [48], a substrate of AKT [49], and a component of mTOR complex 1, and it has been frequently reported to play an important role in tumorigenesis. However, PGK1 phosphorylates PRAS40, inhibits autophagy-mediated cell death and promotes tumorigenesis in early-stage or cancer cells with sufficient oxygen or nutrient supply [20]. Apoptotic cell numbers were notably decreased in PGK1-overexpressing cells but increased in PGK1-depleted cells, and these effects could be reversed by autophagy inhibition. Whether PGK1 can promote tumour occurrence by inducing autophagy in cancer under normal oxygen conditions or under normal nutritional conditions is still under study, and such studies are expected to provide new insights for targeted cancer treatment.

### PGK1 and mitochondrial metabolism

Mitochondria are the most important organelles in the cell and are essential for many cellular processes. As a protein kinase, PGK1 mainly translocates to the mitochondria in response to hypoxic stress. In GBM patients, ERK1/2 phosphorylates PGK1 at Ser203, recruiting PIN1 to mediate Ser203 cis–trans isomerization. Furthermore, the hypoxia-induced activation of EGFR, K-Ras G12V and B-Raf V600E can promote the mitochondrial translocation of PGK1, which leads to the inhibition of mitochondrial pyruvate metabolism and an increase in cytoplasmic glycolysis. Additionally, PGK1 functions as a protein kinase in mitochondria, phosphorylating PDHK1 at Thr338 and reducing PDH-dependent pyruvate metabolism and mitochondrial ROS production [16, 37], thus promoting cell proliferation and tumorigenesis.

### PGK1 and DNA replication

DNA replication is initiated by the interaction of the kinase cell division cycle 7 (CDC7) with its regulatory activation subunit, namely, activator of S-phase kinase (ASK), to activate DNA helicase. EGFR and ERK-activated casein kinase 2a (CK2a) phosphorylate PGK1 at S256 and promote the translocation of a small portion of PGK1 to mitochondria, which results in PGK1 interacting with CDC7. CDC7-bound PGK1 converts CDC7 protein kinase activity-generated ADP to ATP, thereby abrogating the inhibitory effect of ADP on CDC7-ASK activity and promoting the recruitment of phosphorylated MCMs (phosphorylated by CDC7) and DNA helicase to origins of replication; these phenomena ultimately promote DNA replication, cell proliferation, and brain tumorigenesis (Fig. 2D) [50].

PGK1 mediates glycolysis, which generates ATP for tumour cells, especially under hypoxic conditions, and it acts as the gatekeeper of the tricarboxylic acid cycle (TCA cycle), which is related to the development and progression of various cancers [16]. These findings provide new insights into the integrated regulation of glycolysis, autophagy, mitochondrial metabolism and DNA replication, and PGK1 helps promote tumour cell proliferation and maintain cell homeostasis. In brief, the impact of PGK1 on tumour development largely depends on cellular metabolism.

## Molecular mechanisms underlying PGK1 regulation

### Transcriptional regulation

It is well known that the methylation of DNA in gene promoter regions and suppresses the expression of genes at the transcriptional level and is closely associated with carcinogenesis. PGK1 can promote the expression of DNA methylation-related enzymes and DNA repair related proteins through ERK phosphorylation (Fig. 2E). The PGK1 promoter is methylated in multiple types of tumour cells [51]. PGK1 has been shown to be regulated by a series of transcription factors, which regulate the expression of PGK1 through transcription factor networks. In clear cell renal carcinoma cells (ccRCC), Myc is recruited to the promoter region of PGK1, showing that Myc directly interacts with PGK1 [52]. HSP90 was shown to directly interact with PGK1 and exert protumor effects [53]. Additionally, the coil2 domain of KIF15 was shown to bind to PGK1, and KIF15 functions as a scaffold for the direct binding of USP10 and PGK1, which enhances the interaction between USP10 and PGK1 [54]. Additionally, CDC7-bound PGK1 directly converts ADP to ATP, thereby abrogating the inhibitory effect of ADP on CDC7-ASK activity and promoting brain tumorigenesis [50]. Protein‒protein interaction network analysis was also performed and identified 4 hub proteins, namely, PGK1, ALDH5A1, EEF2, and LDHB, that had the highest degrees of connection in adipose tissue. Highly connected proteins are likely to influence the functions of all identified differentially expressed proteins either directly or indirectly [55]. Altogether, these findings implicate PGK1 in rather complex events, which finely modulate the function of PGK1 as either a repressor or activator of gene transcription directly or indirectly.

### Posttranscriptional regulation

Several studies have revealed that ncRNAs function as tumour regulators by targeting transcription factors, including PGK1 (Table 1).

**Table 1:** ncRNAs targeting PGK1 in cancer

Cancer types	ncRNA	Function	Ref
Hepatocellular carcinoma	MiR-450b-3p	PGK1 can be downregulated by the direct binding of miR-450b-3p and inhibits the growth‐promoting effect of miR-450b-3p on HCC	[56]
Glioblastoma	MiR-6869-5p	MiR-6869-5p regulated glioma cell proliferation and invasion via targeting PGK1	[57]
	LncRNA NEAT1	NEAT1 over expression promotes glioma cell proliferation and glycolysis through stabilizing PGK1	[58]
Colorectal cancer	MiR-548c-5p	PGK1 is the target gene of miR-548c-5p, which can influence the proliferation and function of CRC cells by targeting PGK1 and thus plays a cancer-suppressing role	[59]
Breast cancer	MiR-16–1-3p	MiR-16–1-3p inhibits PGK1 expression by directly targeting its 30-untranslated region, and represses breast cancer cell proliferation, migration, invasion, and metastasis	[60]
	LINC00926	FOXO3A/LINC00926/PGK1 axis regulates breast cancer glycolysis, tumor growth, and lung metastasis both in vitro and in vivo	[61]
Prostate cancer	MiR-215-5p	MiR-215-5p alleviates the malignant progression of PCa by targeting and downregulating PGK1	[62]
	MiR-143	Both miR-143 overexpression and curcumin treatment inhibited PGK1 expression and ectopic expression of PGK1 antagonized curcumin’s antitumor effects	[63]
	MiR-29a&MiR-1256	The selective demethylation activity of isoflavone on miR-29a and miR-1256 resulted in the decreased expression of TRIM68 and PGK1, so as to inhibit the growth and invasion mechanism of PCa cells	[64]
	Circ-0076305	Circ-0076305 affects the growth, migration and glycolysis of PCa cells by regulating the miR-411-5p/PGK1 axis	[65]
Lung cancer	MetaLnc9	MetaLnc9 specifically interacted with PGK1, which blocked PGK1 ubiquitination to activate AKT/mTOR signaling pathway	[66]
Tongue squamous cell carcinoma	LncRNA SNHG26	SNHG26 was bind directly to the PGK1 protein, inhibiting its ubiquitination and activating the Akt/mTOR signaling pathway	[67]
Osteosarcoma	LncRNA HCG18	A critical role for HCG18 in the regulation of aerobic glycolysis by sponging miR-365a-3p to elevate PGK1 expression in OS cells	[68]
	Circ-CTNNB1	Circ-CTNNB1 interacted with RBM15 to promoted the expression of hexokinase 2 (HK2), glucose-6-phosphate isomerase (GPI) and phosphoglycerate kinase 1 (PGK1), facilitate the glycolysis process and activate OS progression	[69]

#### MicroRNAs

MicroRNAs (miRNAs) are a group of small, single-stranded ncRNAs (18–25 nucleotides in length) that regulate gene expression by binding to the 3’UTRs of their target mRNAs, and they are involved in tumorigenesis and cancer progression. A growing list of microRNAs (miRNAs) has been shown to regulate PGK1 expression at the posttranscriptional level.

There was a negative correlation between the protein levels of PGK1 and miR-450b-3p in HCC specimens. PGK1 is a direct target of miR-450b-3p, and overexpression of PGK1 can reverse the inhibitory effect of miR-450b-3p on HCC proliferation and cell division [56]. In glioma cells, miR-6869-5p regulates glioma cell proliferation and invasion by targeting PGK1 [57]. In prostate cancer (PCa), PGK1 expression is upregulated, and its expression is negatively regulated by miRNA-215-5p. PGK1 can reverse the regulatory effects of miRNA-215-5p on the metastatic potential of PCa cells. Hence, it is concluded that miRNA-215-5p ameliorates the malignant progression of PCa by targeting and downregulating PGK1 [62]. Another interesting finding from a recent study suggests that miR-143, miR-29a and miR-1256 also perform important biological functions in prostate cancer [63, 64]. In breast cancer patients, miR-16–1-3p inhibits PGK1 expression by directly targeting its 30-untranslated region and represses breast cancer cell proliferation, migration, invasion, and metastasis by inhibiting the PGK1-mediated Warburg effect [60]. In colorectal cancer (CRC), miR-548-5p influences the proliferation and function of CRC cells by targeting PGK1 and thus plays a cancer-suppressing role [59]. In summary, as a common target of various miRNAs, PGK1 can serve as a potential biomarker in cancer cells to influence the occurrence and development of tumours by interacting with miRNAs.

#### lncRNAs

Long noncoding RNAs (lncRNAs), which are usually divided into five categories, are primarily transcribed by RNA polymerase II and lack an obvious open reading frame.

Long noncoding RNA Nuclear paraspeckle assembly transcript 1 (NEAT1) in functionally important for GBM cell growth and metabolic reprogramming. In glioma tissues, lncRNA NEAT1 specifically interacts with PGK1 to block the ubiquitination and degradation of PGK1. LncRNA NEAT1 overexpression promotes glioma cell proliferation and glycolysis by stabilizing PGK1 [58]. In lung cancer, MetaLnc9 promotes the migration and invasion of non-small cell lung carcinoma (NSCLC) cells. Mechanistic research indicated that MetaLnc9 specifically interacts with PGK1, which blocks PGK1 ubiquitination to activate the AKT/mTOR signalling pathway [66]. In breast cancer patients, the FOXO3A/LINC00926/PGK1 axis regulates breast cancer glycolysis, tumour growth, and lung metastasis both in vitro and in vivo [61]. In tongue squamous cell carcinoma (TSCC) cells, SNHG26 directly binds to the PGK1 protein, inhibiting its ubiquitination and activating the Akt/mTOR signalling pathway [67]. In osteosarcoma (OS), lncRNA HCG18 competes for miR-365a-3p with PGK1, which is a key glycolytic coding mRNA, and moderates the repressive effect of miR-365a-3p on PGK1, thereby resulting in increased PGK1 expression and aerobic glycolysis and promoting the proliferation of OS cells [68]. These findings suggest that lncRNAs and PGK1 are involved in the occurrence and development of cancer.

#### CircRNAs

CircRNAs are a subclass of noncoding RNAs characterized by a closed and continuous loop structure without a 5′cap or a 3′poly(A) tail, and they are involved in the pathological processes of multiple malignancies [70]. In OS, circ-CTNNB1 interacts with the m6A regulator RBM15 and subsequently promotes the expression of the key aerobic glycolysis genes hexokinase 2 (HK2), glucose-6-phosphate isomerase (GPI) and PGK1 through m6A modification to facilitate glycolysis and promote OS progression [69]. In prostate cancer, circ_0076305 serves as a novel oncogene by regulating the miR-411-5p/PGK1 axis [65]. However, the precise relationship between PGK1 and circRNAs needs to be further explored.

### Posttranslational regulation

**Fig. 3:**
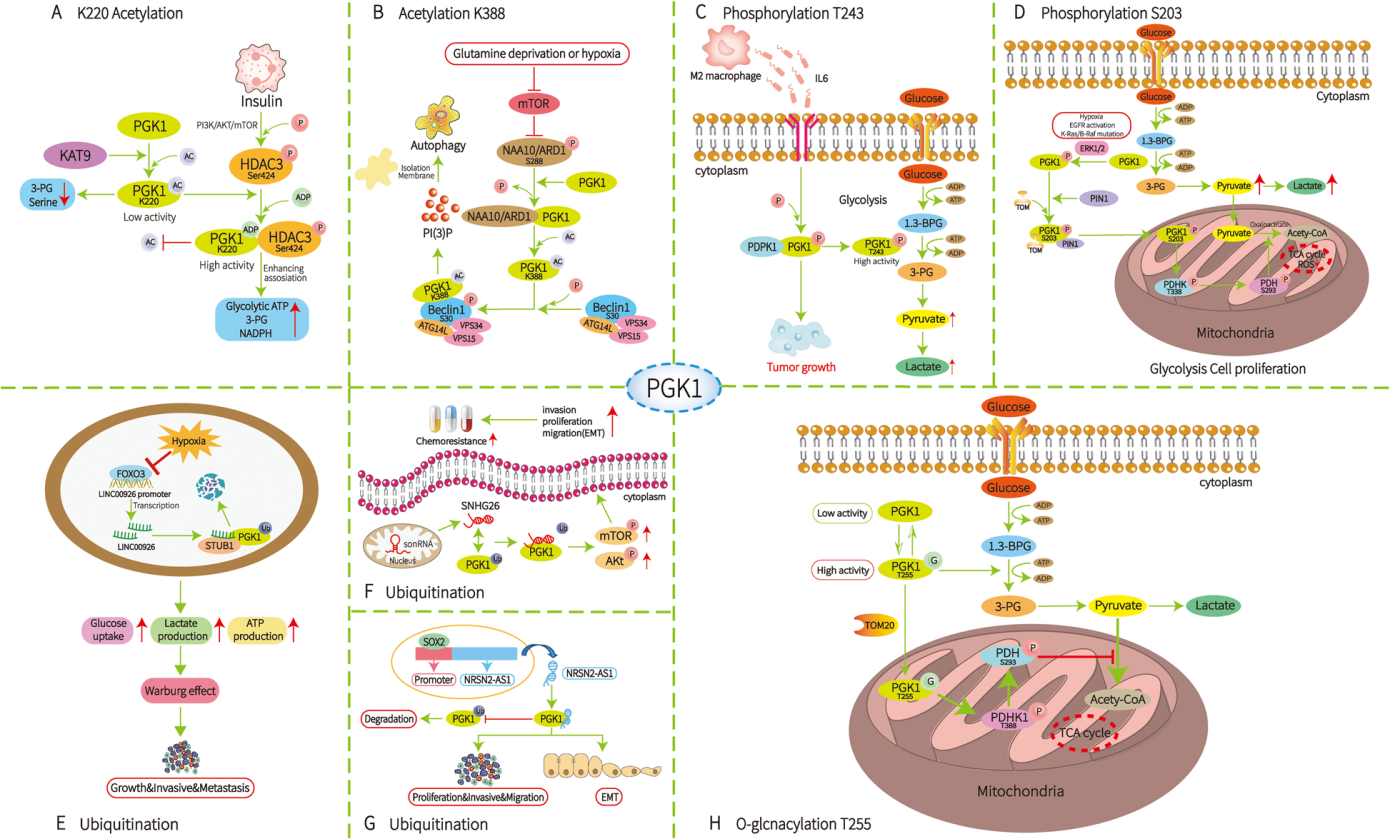
PTM of PGK1, including acetylation, phosphorylation, ubiquitination and O-GlcNAcylation. **A** Under insulin stimulation, PI3K/AKT/mTOR pathway regulates the phosphorylation of HDAC3 Ser424, enhances the interaction between HDAC3 and PGK1, leads to the deacetylation of PGK1 K220, and stimulates the activity/function of PGK1. **B** Glutamine deprivation and hypoxia lead to inhibition of mTOR-mediated phosphorylation of NAA10/ARD1 S228, leading to PGK1 K388 acetylation and Beclin1 S30 phosphorylation, which activates the autophagosome to form the required complex. **C** Polarized M2 macrophages secrete IL-6 to increase PDPK1-dependent PGK1 phosphorylation at T243 in GBM cells. **D** The phosphorylation of PGK1 at serine 203 (S203) correlates with the promotion of the Warburg effect, hypoxia, expression of oncogenic genes, and cancer development. **E** FOXO3A/LINC00926/PGK1 axis regulates breast cancer glycolysis, tumor growth, and lung metastasis both in vitro and in vivo. **F** SNHG26 binds to PGK1 protein, inhibits its ubiquitination and activates the Akt/mTOR signaling pathway. **G** NRSN2-AS1, transcribed by SOX2, exerts its tumor promoting activity by binding to PGK1. **H** PGK1 is dynamically modified reversely by O-GlcNAc at the threonine 255 (T255) site, enhancing PGK1 activity while inducing PGK1 translocation to mitochondria. Ac, acetylation; P, phosphorylation; Ub, ubiquitination; G, O-GlcNAcylation

PTMs are important mechanisms that regulate the functions of proteins and alter their stability, subcellular location, transcriptional activity and interaction with other biomolecules. The most common PTMs include phosphorylation, acetylation, glycosylation, methylation, ubiquitination and sumoylation. These site-specific modifications are differentially regulated by various stimuli or the tumour microenvironment. Accumulating evidence reveals that the functions of PGK1 are altered by several PTMs, which can influence their target gene expression (Fig. 3).

#### Acetylation of PGK1

Acetylation is a reversible, highly dynamic modification that neutralizes and reduces the charge of lysine residues and modifies protein structures, thus affecting DNA-binding affinity, enzymatic activity, protein stability, and subcellular localization [71]. A recent study showed that lysine 220 (K220) is an important regulatory acetylation site within the PGK1 protein. K220 acetylation plays an important role in regulating PGK1 activity and/or function to modulate glycolytic ATP production, glucose metabolism and redox status. Studies [11] have proven that upon insulin stimulation, the PI3K/AKT/mTOR pathway regulates HDAC3 phosphorylation at Ser424, which increases the deacetylase activity of HDAC3 and enhances the interaction between the overexpressed HDAC3 and PGK1 proteins, leading to PGK1 K220 deacetylation and stimulating PGK1 activity/function. Whether the occurrence and development of tumours are related to the K220 acetylation of PGK1 needs to be further studied.

Recent studies [72] have found that glutamine deprivation and hypoxia lead to inhibition of mTOR-mediated NAA10/ARD1 S228 phosphorylation, resulting in NAA10/ARD1-dependent PGK1 K388 acetylation and subsequent PGK1-mediated Beclin1 S30 phosphorylation, which activates the VPS34-Beclin1-ATG14L complex that is required for autophagosome formation. More importantly, the level of PGK1 acetylation at K388 is positively correlated with the level of phosphorylated Beclin1 S30 and is associated with poor prognosis in GBM patients [17]. GBM patients with high PGK1 K388 acetylation levels have a significantly shorter survival than those with low PGK1 K388 acetylation levels.

#### Phosphorylation of PGK1

Protein phosphorylation is one of the most common PTMs as well as an important cellular regulatory mechanism because many enzymes and receptors are activated/deactivated via phosphorylation and dephosphorylation events that are mediated by kinases and phosphatases [73]. Zhang et al.[23] demonstrated that polarized M2 macrophages secrete IL-6 to increase PDPK1-dependent phosphorylation of PGK1 at T243 in GBM cells and promote glycolysis. In addition, T243 phosphorylation of PGK1 is associated with macrophage infiltration, grading and prognosis of GBM patients [23]. This is a novel mechanism by which macrophages promote tumour growth by regulating the metabolism of tumour cells.

Another report showed that phosphorylation of PGK1 at serine 203 (S203) correlates with increases in the Warburg effect, hypoxia, expression of oncogenic genes, and cancer development. Multiple factors, including hypoxia, EGFR activation, and K-Ras/B-Raf mutation, can induce PGK1 phosphorylation at S203 by extracellular signal-regulated kinase 1/2 (ERK1/2) [16]. In the mitochondria, PGK1 functions as a protein kinase to phosphorylate and activate PDHK1 at T338, which further leads to phosphorylation and inactivation of the pyruvate dehydrogenase (PDH) complex [16, 17]. In conclusion, phosphorylation of PGK1 at various sites may be an attractive approach for cancer treatment.

#### Ubiquitination of PGK1

Ubiquitination, which generally promotes protein degradation, plays a critical role in both physiological and pathological processes, such as reproduction, growth and development, signal transduction, and tumorigenesis [74, 75]. Zhong chu [61] demonstrated that LINC00926 interacts with PGK1 and promotes its ubiquitination and degradation by binding to the E3 ligase STUB1, which is the first E3 ligase responsible for PGK1 degradation and may be a potential therapeutic target for breast cancer. Jiang et al. [67] performed mechanistic investigations showing that SNHG26 can directly bind to PGK1 protein, inhibit its ubiquitination, and activate the Akt/mTOR signalling pathway in tongue squamous cell carcinoma (TSCC). Xu et al. [76] showed that NRSN2-AS1 exerts its regulatory function by interacting with PGK1 protein in esophageal cancer and regulates its stability by inhibiting its ubiquitination.

In conclusion, promoting the degradation of PGK1 by increasing its ubiquitination may be an effective strategy for PGK1-targeted cancer therapies.

#### O-GlcNAcylation of PGK1

GlcNAc is a prevalent PTM that is added to serine and threonine residues in proteins. The addition and removal of O-GlcNAc in protein substrates are mediated by two enzymes, O-GlcNAc transferase (OGT) and O-GlcNAc hydrolase (OGA), respectively [77, 78]. PGK1, the first ATP-producing enzyme in glycolysis, is reversibly and dynamically modified with O-GlcNAc at threonine 255 (T255), and this site-specific glycosylation enhances PGK1 activity and simultaneously induces PGK1 translocation to the mitochondria. In the mitochondria, PGK1 acts as a kinase to inhibit the pyruvate dehydrogenase (PDH) complex to reduce pyruvate utilization and suppress oxidative phosphorylation [21].

With the development of multiple technologies, more PTMs of PGK1 may be revealed to regulate cell metabolism and growth. Increasing attention will be given to the interplay among different PTMs. This will facilitate the design of drugs to interfere with tumorigenesis and cancer progression.

## PGK1 and its clinical significance in cancer

**Fig. 4:**
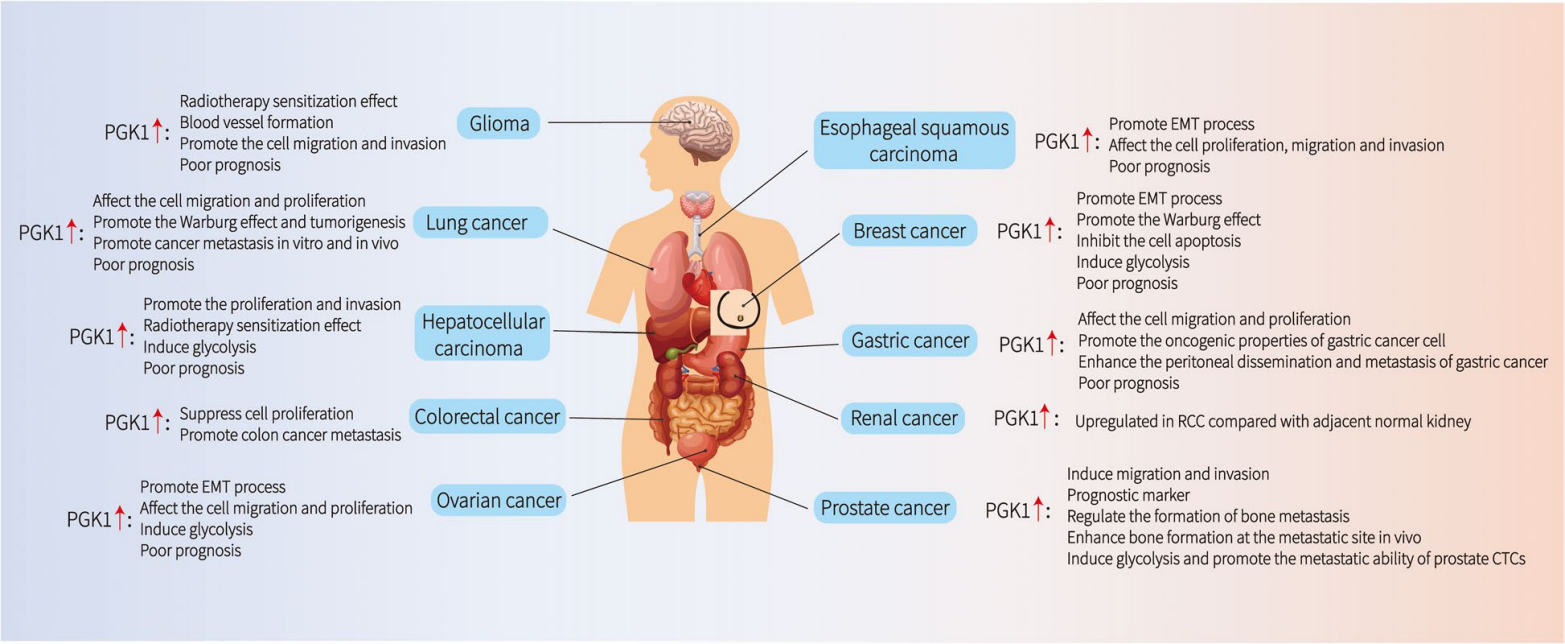
Correlation of PGK1 levels with prognosis of cancer patients. In most human cancers, PGK1 overexpression or amplification is associated with poor survival of cancer patients

Given the critical role of PGK1 in the initiation and progression of tumorigenesis, it is not surprising that PGK1 dysregulation frequently occurs in human cancer tissues. Indeed, PGK1 overexpression or amplification is often observed in many different types of human cancers, which, importantly, is correlated with poor survival of cancer patients (Fig. 4).

In Chinese patients with endometrial cancer, immunohistochemical analysis revealed increased PGK1 expression in the cytoplasm of endometrial carcinoma cells compared with that in normal endometrial tissues [79]. PGK1 is also overexpressed in lung adenocarcinoma (LUAD) and serves as an independent poor prognostic marker. It has been reported that PGK1 is an independent prognostic indicator of hepatocellular carcinoma (HCC) and may be a new therapeutic target for HCC. PGK1 might also be an independent factor for the recurrence of HCC [80]. The overexpression of PGK1 and its signalling targets may be an expression regulatory pathway that promotes peritoneal dissemination in diffuse primary gastric carcinomas, and these factors may function as prognostic markers and/or be potential therapeutic targets to prevent the migration of gastric carcinoma cells into the peritoneum [81]. In gallbladder cancer (GBC) samples, PGK1 is significantly downregulated compared with the control samples and is associated with multiple clinicopathological factors. Multivariate survival analysis showed that low PGK1 expression is associated with shorter OS and DFS, indicating that PGK1 expression is an independent prognostic factor in patients with GBC [82]. Specifically, in HNSCC, PGK1 overexpression is frequently observed in primary tumours but not in healthy tissues [30], and elevated expression of PGK1 is significantly correlated with poor prognosis [83].

However, there are several challenges in the use of PGK1 as a clinical biomarker for the diagnosis, prognosis and treatment of various types of cancers. First, the exact relationship between the expression levels of PGK1 and the degree of tumour progression has not yet been established. Second, the correlation between PGK1 expression and different subtypes of tumours requires further clarification. Thus, larger population-based studies are still needed to further confirm the utilization of PGK1 as a biomarker in cancer.

## PGK1-targeting approaches

**Fig. 5:**
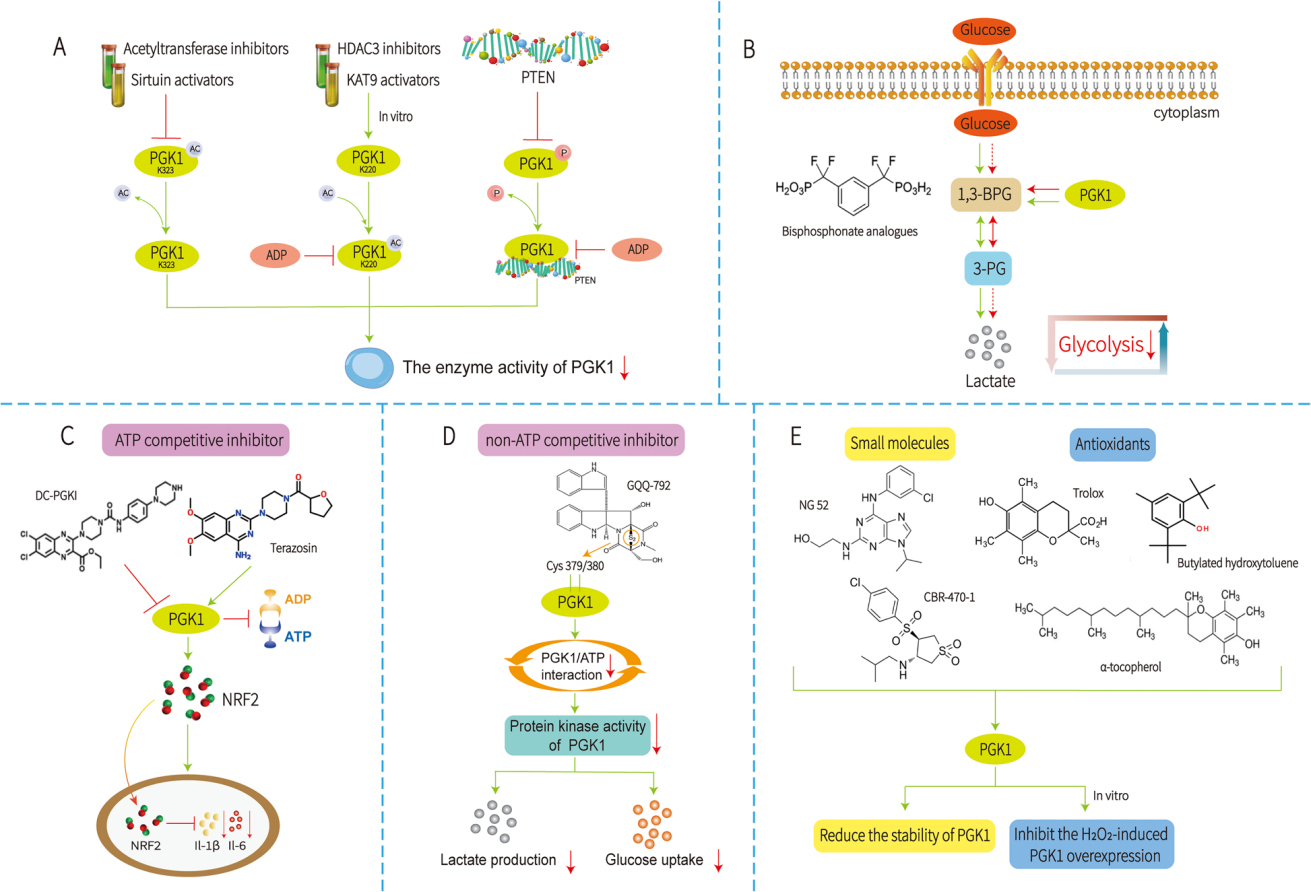
Potential inhibitors for PGK1. **A** PGK1 enzyme activity was inhibited by regulating ptm. **B** Bisphosphate analogues of 1, 3-bpg. **C** ATP competition inhibitor. **D** Non-ATP competition inhibitor. E. Small molecules & Antioxidants

For most human cancers, PGK1 acts as an oncoprotein by activating several proliferative and antiapoptotic signalling cascades to promote tumorigenesis, metastasis, and drug resistance. Targeting PGK1 is, therefore, an effective strategy for anticancer therapy. Due to the lack of promising lead compounds, research on small molecule inhibitors of PGK1 has been very limited to date [7], although several PGK1-targeting approaches have been investigated (Fig. 5).

The first approach is to inhibit the enzymatic activity of PGK1 by modulating PTMs. For example, K323 acetylation of PGK1 is thought to promote the enzymatic activity of PGK1; therefore, the use of acetyltransferase inhibitors or sirtuin activators can inhibit the activity of PGK1 [7]. Similarly, a KAT9 activator or HDAC3 inhibitor can inhibit the level of PGK1K220 acetylation. When the interaction between PGK1 and its substrate ADP is blocked, the activity of PGK1 is inhibited [11]. PTEN directly interacts with PGK1 to phosphorylate and inhibit PGK1 activity, thus acting as a protein phosphorylase. PTEN inhibits PGK1 autophosphorylation, glycolysis, and ATP production and controls cell proliferation and tumorigenesis. Inhibition of PGK1 autophosphorylation prevents glycolysis and brain tumour development [84]. However, the specificity of these indirect approaches is controversial, mainly because PGK1 is not the only target of these inhibitors.

The second approach focuses on analogues of the natural substrate 1,3-BPG. Bisphosphonate analogues of 1,3-BPG are one of the earliest synthesized PGK1 competitive inhibitors. These bisphosphates can competitively bind to PGK1, which weakens PGK1 binding to 1,3-BPG, thus reducing the activity of PGK1 [85]. However, the effects of these PGK1 inhibitors on cancer cells have not been reported.

The third approach is an ATP competitive inhibitor. LTP-10, with a new chemical scaffold, is an ATP competitive inhibitor of PGK1 [86]. DC-PGKI is an ATP competitive inhibitor of PGK1 with a high binding affinity; DC-PGKI inhibits PGK1 and suppresses the production of Il-1β and IL-6 via the accumulation of NRF2, which translocates to the nucleus and then binds to the proximity of the IL-1β and IL-6 genes [86]. Moreover, terazosin was also considered a potential competitive inhibitor of PGK1 [85]. However, the therapeutic application of such an approach is debatable.

The fourth approach is to use non-ATP competitive inhibitors. GQQ-792 is a novel type of epipolythiodiketopiperazine (EPT) isolated from Tilachlidium sp. mangrove endophytic fungus. It inhibits the enzymatic activity of PGK1, a kinase involved in glycolysis, by binding to a cysteine residue near the ATP-binding pocket of PGK1 through its disulfide group as a pharmacophore. GQQ-792 is a non-ATP-competitive inhibitor and exhibits antitumour activity in vitro and in vivo by directly binding to PGK1 and inhibiting its kinase activity in U87-MG glioblastoma cells [87].

The fifth approach is to use small molecules that regulate PGK1 or are regulated by PGK1 to indirectly inhibit PGK1. For example, NG52 can reverse the Warburg effect by inhibiting PGK1 kinase activity and switching cellular glucose metabolism from an anaerobic mechanism to an aerobic mechanism, thereby inhibiting the growth of tumour cells [88]. Moreover, when induced by hydrogen peroxide, several antioxidants, including α-tocopherol, butylated hydroxytoluene and Trolox, can reverse the increase in PGK1 expression [42]. Recently, a small-molecule compound, CBR-470–1, was identified as a new novel inhibitor of PGK1 [89]. However, the specific mechanism by which CBR-470–1 inhibits PGK1 is unclear.

In recent years, great progress has been made in the application of PGK1 in clinical treatment. In 2022, Helena Chaytow et al. studied the efficacy of targeting PGk1 using terazosin in zebrafish, mouse and ESC-derived motor neuron models of ALS [90]. Then, we retrieved a clinical trial named Terazosin RepUrposing Study in amyotrophic lateral sclerosis: a pilot study targeting PGK1 with terazosin in ALS patients (ID: ISRCTN45028842) from ICTRP Search Portal (who.int). In addition, in the clinical trial Polymorphisms in key genes of glycolytic pathway: Influence on radiosensitivity in nasopharyngeal carcinoma (ID: NCT02481089) (https://clinicaltrials.gov/), investigators discovered that glycolysis-related genes such as PGK1 and ALDOA were associated with radiosensitivity. However, there is still a long way to go before new PGK1 inhibitors can be developed for clinical treatment.

## Conclusion and perspectives

In the present review, the biological functions of PGK1 were introduced, with a focus on its influence on cancer. We found that in mitochondria, PGK1 can function as a protein kinase to inhibit pyruvate metabolism and promote the Warburg effect. In the nucleus, PGK can increase the synthesis of DNA. In addition, PGK1 also plays an important role in tumour energy metabolism. However, the role of PGK1 in tumours is different, which may be related to the tissue specificity of PGK1 and its expression level in different tissues; this should be considered when developing drugs and tumour treatments that target PGK1. In addition, developing therapeutic drugs that target PGK1 according to the function of PGK1 is also an important issue. Based on these unresolved discrepancies, the authors' prediction is that the future exploration of PGK1 will focus on the following topics. First, PGK1 and other metabolic pathways are intertwined, and it is necessary to understand the functional consequences of targeting this enzyme in cancer cells. Second, future strategies for developing PGK1 inhibitors may include virtual screening (i.e., similarity search, pharmacophore model, shape-based model and molecular docking) based on the X-ray structure of the PGK1/terazosin complex. Third, designing strong inhibitors of PGK1 and then seeking to modify and optimize them will also be trend of future research. Additionally, to ensure that therapeutic or diagnostic strategies based on PGK1 are safe for patients with cancer, all applications should be carefully considered with sufficient scientific research.
